# Genetic Diversity and Population Structure Reveal Cryptic Genetic Variation and Long Distance Migration of *Puccinia graminis* f. sp. *tritici* in the Indian Subcontinent

**DOI:** 10.3389/fmicb.2022.842106

**Published:** 2022-04-13

**Authors:** Pramod Prasad, Rajni Kant Thakur, Siddanna Savadi, Subhash Chander Bhardwaj, Om Prakash Gangwar, Charu Lata, Sneha Adhikari, Subodh Kumar

**Affiliations:** ^1^ICAR-Indian Institute of Wheat and Barley Research, Regional Station, Shimla, India; ^2^ICAR-Directorate of Cashew Research, Puttur, India

**Keywords:** wheat, stem rust, virulence phenotype, SSR genotype, genetic diversity, evolution

## Abstract

Stem rust caused by *Puccinia graminis* f. sp. *tritici* (*Pgt*) is a devastating disease of wheat worldwide since time immemorial. Several wheat stem rust outbreaks have been reported worldwide including India. Approximately 7 mha wheat area in central and peninsular India is highly vulnerable to stem rust epidemics. In this study, a repository of 29 single genotype uredospore pathotypes, representing five geographical regions, was characterized by investigating their virulence phenotype and simple sequence repeat (SSR) genotypes using 37 reproducible polymorphic SSR markers, 32 of which had ≥ 0.50 polymorphic information content (PIC) value. Virulence phenotypes were used to evaluate the virulence frequency (VF) and construct a hypothetical evolutionary hierarchy of these pathotypes. We projected seven lineages to explain the evolutionary pattern of the *Pgt* population. The VF of these pathotypes ranged between 0% and 100%. The virulence-based neighbor-joining (NJ) cluster analysis grouped *Pgt* pathotypes into five virulence groups. Likewise, five molecular groups were categorized using molecular genotypes. The molecular grouping was supported by principal coordinate analysis (PCoA), which revealed 25% of the cumulative variance contributed by the first two axes. Analysis of molecular variance (AMOVA) revealed 8 and 92% of the variation among and within the populations, respectively. The Mantel test confirmed a positive but weak correlation (*R*^2^ = 0.15) between virulence phenotypes and SSR genotypes. The highest and lowest values of different genetic diversity parameters (Na, Ne, I, He, uHe, and %P) revealed maximum and minimum variability in the *Pgt* population from Maharashtra and Uttar Pradesh, respectively. The population structure analysis clustered 29 *Pgt* pathotypes into two subpopulations and an admixture. Our results demonstrated that there was significant genetic diversity among *Pgt* pathotypes resulting from their long-distance dispersal ability complemented by gene flow. These findings provide insights into the virulence patterns, genetic variations, and possible evolution of *Pgt* pathotypes, which would support strategic stem rust resistance breeding.

## Introduction

Wheat (*Triticum aestivum* L.) is one of the most important sources of human nutrition and plays a critical role in global food security. The global wheat production reached 760.7 million tons during 2019, and 31% of it came from the two Asian countries, i.e., China and India, ranked first and second in global wheat production, respectively (FAO, 2021). Wheat production has been continuously threatened by several abiotic and biotic factors including diseases. Stem (black) rust caused by *Puccinia graminis* f. sp. *tritici* Eriks. and Henn. *(Pgt)* is a highly devastating disease of wheat and could result in wheat yield losses up to 100% on susceptible wheat cultivars (Anonymous, 1982; Worku et al., 2016). It is an obligate biotrophic, macrocyclic, and heteroecious fungus. The *Pgt* population comprises pathotypes with a highly diverse virulence nature or in some cases could be extremely uniform, which can disperse over long distances through wind and evolve new pathotypes that could overcome the stem rust resistance in wheat cultivars (Singh et al., 2015; Prasad et al., 2016).

Stem rust epidemics have occurred in the majority of wheat-growing regions of the world including East Africa, Eastern Europe, Australia, North America, Canada, the United States, and India (Dill-Macky et al., 1990; Eversmeyer and Kramer, 2000; Prasad et al., 2017b,^2020^). In India, stem rust is known to affect wheat crops in approximately 7 Mha areas covering the Central and Southern parts of the country. Historically, severe stem rust outbreak on Einkorn wheat was reported during 1907, followed by epidemics of 1946–1947 and 1948–1949 in Central and Southern India, respectively (Mehta, 1952; Prasad et al., 2018). With the help of strong disease surveillance, availability of suitable stem rust-resistant wheat cultivars, and high varietal replacement rate, the rusts have been kept under check; and no wheat rust epidemics were reported from any of the wheat-growing areas from India in the post green revolution period. Mehta (1952) reported a total loss of approximately 60 million Indian rupees annually in wheat and barley due to rust diseases in India. Stem rust resistance genes *Sr2, Sr5, Sr6, Sr7a, Sr7b, Sr8a, Sr8b, Sr9b, Sr9e, Sr11, Sr12, Sr13, Sr17, Sr21, Sr24, Sr25, Sr30*, and *Sr31* are very common in Indian wheat cultivars. Among these, *Sr2, Sr11*, and *Sr31* are more frequent in bread wheat, whereas *Sr7b, Sr9e, Sr11*, and *Sr13* have been postulated in many durum cultivars (Jain et al., 2013). The emergence of TTKSK (also known as Ug99), one of the most virulent forms of *Pgt*, was first identified from Uganda in 1998 (Pretorius et al., 2000) and its variants (15 races), reported in 14 countries including Egypt, Yemen, and Iran (Singh et al., 2015; https://rusttracker.cimmyt.org/?page_id=22) and has rendered 90% of the world wheat varieties susceptible to stem rust (Singh et al., 2015; Worku et al., 2016; Soko et al., 2018). Another stem rust race, namely, Digalu (TKTTF) caused 100% yield loss in many Ethiopian regions during 2013–2014 and 2014–2015 (Olivera et al., 2015). Thus, stem rust has become a massive menace for world wheat production and has threatened global food security (Patpour et al., 2016; Prasad et al., 2016; Olivera et al., 2019).

The stem rust management in wheat primarily relies on the cultivation of disease-resistant varieties in combination with appropriate agronomic practices and the use of fungicides (Bhardwaj et al., 2016; Savadi et al., 2017). However, race-specific resistance is often overcome by the frequent introduction or evolution of new *Pgt* pathotypes through mutation, somatic recombination, or sexual recombination (Patpour et al., 2016). Therefore, regular monitoring of variations in the *Pgt* populations is essential for the identification of new resistance sources and anticipatory breeding for rust resistance to effectively manage the newly emerging *Pgt* isolates. The understanding of the virulence and genetic diversity, distribution, and population structure of the *Pgt* population allows effective monitoring of the emerging stem rust challenge, which is critical in formulating wheat stem rust management strategies (Abebe et al., 2012).

Virulence-based phenotyping of *Pgt* races in India started way back in 1932, and since then, 29 pathotypes of *Pgt* have been identified from the Indian subcontinent based on their pathogenicity on Indian stem rust differentials (Prasad et al., 2018). However, the genetic analysis of these pathotypes using molecular markers is not reported till date. Genetic analysis and genotypic variability of plant-pathogen populations have assisted researchers to reveal the genetic relationship among different pathotypes and track the pathways by which these pathogens evolve over time and space (Olivera et al., 2015; Prasad et al., 2017a). The simple sequence repeat (SSR) or microsatellite markers are most extensively used and established for exploring population dynamics and evolutionary patterns of *Puccinia* spp. due to the multi-allelic, reproducible, and highly polymorphic nature of these markers (Zhong et al., 2009; Savadi et al., 2020). This study was conducted to explore the virulence-based phenotype and SSR marker-based genotype patterns, population structure, and other diversity parameters in a repository of 29 *Pgt* pathotypes collected during the past ∼90 years from India and neighboring countries and maintained *in vivo* at ICAR-Indian Institute of Wheat and Barley Research, Regional Station (ICAR-IIWBR, RS), Shimla, India. Every year 100–200 isolates of *Pgt* collected from different wheat stem rust-prone areas in the Indian subcontinent are analyzed. Since the alternate host for stem rust is not functional, only 29 pathotypes have been identified in *Pgt* from this region. These pathotypes have been identified from different locations and years. The information on the distribution of these pathotypes during different years and locations is being published regularly, and the latest was by Prasad et al. (2018).

## Materials and Methods

### Purification and Multiplication of *Puccinia graminis tritici* Pathotypes

**FIGURE 1:**
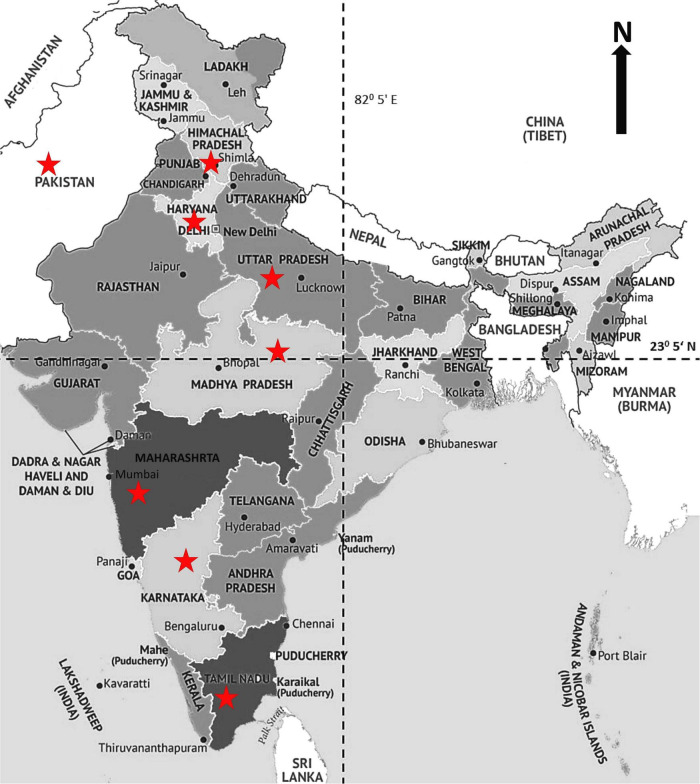
Wheat growing areas of India showing the field locations (indicated by a star), where *Puccinia graminis* f. sp. *tritici* pathotypes were initially detected.

Twenty-nine *P. graminis* f. sp. *tritici* (*Pgt*) pathotypes, detected from the Indian subcontinent (Figure 1) and maintained at ICAR-IIWBR, RS, Shimla, were selected to study their virulence-based phenotype and SSR marker-based genotype patterns (Table 1). Single pustules, isolated for all the pathotypes to minimize the chances of heterogeneity within individual pathotypes, were multiplied on susceptible wheat variety “Agra local.” The uredospores from all the pathotypes were collected and stored for purity check, virulence phenotype, and SSR genotype analysis.

**TABLE 1:** Designation, geographic origin, year of identification, and avirulence/virulence formula of 29 pathotypes of *Puccinia graminis* f. sp. *tritici* from the Indian subcontinent used to study virulence phenotype and simple sequence repeat (SSR) genotypes.

S. no.	Designation			First detection		Avirulence/virulence formula
	New	Old	North American Equivalent*	Year	Place	
1	79G31	11	RRTSF	1962	Maharashtra	*Sr7a, 8a, 8b, 9e, 22, 23, 24, 25, 26, 27, 31, 32, 33, 35, 37, 39, 40, 43, Tmp, Tt3*/*5, 6, 7b 9a, 9b, 9c, 9d, 9f, 9g, 10, 11, 13, 14, 15, 16, 17, 18, 19, 20, 21, 28, 29, 30, 34, 36, 38, McN*
2	203G15	11A	RHTSF	1974	Wellington, Tamil Nadu	*Sr7a, 8a, 8b, 9e, 11, 22, 23, 24, 25, 26, 27, 31, 32, 33, 35, 39, 40, 43, Tmp, Tt3*/*5, 6, 7b 9a, 9b, 9c, 9d, 9f, 9g, 10, 12, 13, 14, 15, 16, 17, 18, 19, 20, 21, 28, 29, 30, 34, 36, 37, 38, McN*
3	16G2	14	GKBSC	1959	Gwalior, Madhya Pradesh	*Sr5, 7a, 7b, 9b, 9e, 11, 13, 17, 22, 23, 24, 25, 26, 27, 28, 29, 30, 31, 32, 33, 34, 35, 36, 37, 39, 39, 40, 43, Tmp, Tt3*/*2, 6, 8a, 8b, 9a, 9d, 9f, 9g, 10, 14, 15, 16, 18, 19, 20, 21, McN*
4	123G15	15-1	TKTSF	2008	Karnataka	*Sr7a, 11, 24, 25, 26, 27, 31, 32, 33, 35, 37, 39, 40, 43, Gt, Tmp, Tt3*/*5, 6, 7b, 8a, 8b, 9a, 9b, 9d, 9e, 9f, 9g, 10, 12, 13, 14, 15, 16, 17, 18, 19, 20, 21, 22, 23, 28, 29, 30, 34, 36, 38, 42, 44, McN, Wld*
5	9G5	21	CHMQC	1935	Lyallpur, Pakistan, Himachal Pradesh	*Sr5, 7a, 8a, 8b, 9b, 9c, 9e, 10, 11, 12, 15, 21, 22, 23, 24, 25, 26, 27, 29, 30, 31, 32, 33, 34, 35, 37, 38, 39, 40, 43, Gt, Tmp, Tt3*/*6,7b, 9d, 9f, 9g, 13, 14, 16, 17, 19, 28, 36, McN*
6	24G5	21-1	CKMSC	1985	Uttar Pradesh	*Sr5, 7a, 9b, 9e, 11, 13, 21, 23, 24, 25, 26, 27, 29, 30, 31, 32, 33, 35, 37, 38, 39, 40, 43, Tmp, Tt3*/*6, 7b, 8a, 8b, 9a, 9d, 9f, 9g, 10, 14, 15, 16, 17, 18, 19, 20, 22, 28, 34, 36, McN*
7	75G5	21A-2	CCTJC	1962	Indore, Madhya Pradesh	*Sr5, 6, 7a, 8a, 8b, 9a, 9c, 9e, 11, 12, 21, 22, 23, 24, 25, 26, 27, 29, 31, 32, 33, 35, 37, 38, 39, 40, 43, Gt, Tmp, Tt3*/*7b, 9b, 9d, 9f, 9g, 10, 13, 14, 15, 16, 17, 19, 28, 30, 34, 36, McN*
8	5G19	24A	HRMSF	1981	Powarkheda, Madhya Pradesh	*Sr5, 7a, 8a, 8b, 9b, 9e, 24, 25, 26, 27, 28, 30, 31, 32, 33, 35, 36, 37, 39, 40, 43, Tmp*/*2, 6, 7b, 9a, 9d, 9f, 9g, 10, 11, 12, 13, 14, 15, 16, 17, 18, 19, 20, 21, 22, 23, 29, 34, 36, 38, 42, 44, McN*
9	10G13	34-1	MCGGP	1991	Gotegaon, Madhya Pradesh	*Sr6, 7a, 8a, 8b, 9a, 9e, 10, 11, 13, 17, 19, 21, 22, 23, 24, 25, 26, 27, 30, 31, 32, 33, 35, 36, 37, 39, 40,43, Tmp, Tt3*/*5, 7b, 9b, 9d, 9f, 9g, 14, 15, 16, 18, 20, 28, 29, 34, 38, McN*
10	104G13	40	PHDGC	1932	Pune, Maharashtra	*Sr2, 7a, 8a, 8b, 9a, 9b, 9c, 10, 11, 12, 13, 14, 17, 21, 22, 23, 24, 25, 26, 27, 29, 31, 32, 33, 35, 36, 37, 38, 39, 40, 43, Tmp, Tt3/5, 6, 7b, 9d, 9e, 9f, 15, 16, 19, 28,30, 34, McN*
11	62G29	40A	PTHSC	1974	Wellington, Tamil Nadu	*Sr7a, 13, 21, 22, 24, 25, 26, 27, 30, 31, 32, 33, 35, 36, 37, 38, 39, 40, 43, Tmp, Tt3*/*5, 6, 7b, 8a, 8b, 9a, 9b, 9d, 9e, 9f, 9g, 10, 11, 14, 15, 16, 17, 18, 19, 20, 23, 28, 29, 34, McN*
12	62G29-1	40-1	PTHSM	1989	Wellington, Tamil Nadu	*Sr7a, 13, 21, 22, 25, 26, 27, 30, 31, 32, 33, 35, 36, 37, 38, 39, 40, 43, Tmp, Tt3*/*5, 6, 7b, 8a, 8b, 9a, 9b, 9d, 9e, 9f, 9g, 10, 11, 14, 15, 16, 17, 18, 19, 20, 23, 24, 28, 29, 34, McN*
13	58G13-3	40-2	PKRSC	2006	Karnataka	*Sr7a, 11, 13, 14, 21, 22, 23, 24, 26, 27, 29, 30, 31, 32, 33, 35, 37, 38, 39, 40, 43, Tmp*/*5, 6, 7b, 8a, 8b, 9a, 9b, 9d, 9e, 9f, 9g, 10, 12, 15, 16, 17, 18, 19, 20, 25, 28, 34, 36, 42, Wld-1, McN, Gt*
14	127G29	40-3	PTKSF	2008	Dharwad, Karnataka	*Sr21, 22,24, 25, 26, 27, 31, 32, 33, 35, 36, 37, 39, 40, 42, 43, Tmp, Tt3*/*5, 6, 7a, 7b, 8a, 8b, 9a, 9b, 9d, 9e, 9f, 9g, 10, 11, 14, 15, 16, 17, 18, 19, 20, 23, 28, 29, 30, 34, 38, 44, McN, Gt*
15	19G35	42	HKGGC	1932	Pune, Maharashtra	*Sr2, 5, 9a, 9c, 9e, 10, 11, 17, 22, 23, 24, 25, 26, 27, 28, 29, 30, 31, 32, 33, 34,35, 36, 37, 38, 39, 40, 43, Tmp, Tt3/6, 7a, 7b, 8a, 8b, 9b, 9d, 9f, 12, 13, 14, 15, 16, 19, 21, McN*
16	7G35	42B	HRHJC	1947	Mahabaleshwar, Maharashtra	*Sr2, 5, 8a, 8b,9a, 9c, 9e, 22, 24, 25, 26, 27, 28, 29, 30, 31, 32, 35, 36, 37, 38, 39, 40, 43, Tmp, Tt3/6, 7a, 7b, 9b, 9d, 9f, 10, 11, 12, 13, 14, 15, 16, 17, 19, 21, 23, 33, 34, McN*
17	37G3	117	KRCSC	1945	Vetool, Karnataka	*Sr7a, 5, 8, 9b, 12, 22, 24, 25, 26, 27, 28, 30, 31, 32, 33, 35, 36, 37/6, 7b, 9a, 9c, 9d, 9e, 9f, 10, 11, 13, 14, 15, 16, 17, 19, 21, 23, 29, 34*
18	36G2	117A	KRCQC	1961	Karnataka	*Sr5, 8, 9b, 10, 12, 13, 14, 16, 22, 24, 25, 26, 27, 28, 30, 31, 32, 33, 36, 37/6, 7b, 9a, 9c, 9d, 9e, 9f, 11, 15, 21, 23, 29, 34*
19	38G18	117A-1	HRHSC	1977	Dharwad, Karnataka	*Sr5, 7b, 8, 12, 13, 22, 24, 25, 26, 27, 28, 30, 31, 32, 33, 35, 36, 37/6, 7a, 9a, 9b, 9c, 9d, 9e, 9f, 10, 11, 14, 15, 16, 17, 19, 21, 23, 29, 34*
20	166G2	117-1	JRHSC	1987	Dharwad, Karnataka	*Sr5, 7a, 7b, 8, 12, 13, 14, 22, 24, 25, 26, 27, 28, 30, 31, 32, 33, 35, 36/6, 9a, 9b, 9c, 9d, 9e, 9f, 10, 11, 15, 16, 17, 19, 21, 23, 29, 34, 37*
21	33G3	117-2	KHCSC	1987	Karnal, Haryana	*Sr5, 7a, 8a, 8b, 9b, 11, 12, 22, 24, 25, 26, 27, 28, 30, 31, 32, 33, 35, 36, 37, Tmp*/*2, 6, 7b, 9a, 9c, 9d, 9e, 9f, 9g, 10, 13, 14, 15, 16, 17, 19, 21, 23, 29, 34, McN*
22	167G3	117-3	KRCSC	1987	Wellington, Tamil Nadu	*Sr5, 8a, 8b, 9b, 22, 24, 25, 26, 27, 28, 30, 31, 32, 33, 35, 36, 38, 39, 40, 43, Tmp*/*2, 6, 7a, 7b, 9e, 9f, 9g, 10, 11, 12, 13, 14, 15, 16, 17, 19, 21, 23, 29, 34, 37, McN*
23	166G3	117-4	KMGSC	1987	Dharwad, Karnataka	*Sr5, 6, 7a, 8a, 8b, 9c, 9f, 12, 13, 14, 17, 19, 22, 23, 24, 25, 26, 27, 28, 30, 31, 32, 33, 35, 36, 38, 39, 40, 43, Tmp/2, 7b, 9a, 9b, 9e, 9d, 9e, 9g, 10, 11, 15, 16, 21, 29, 34, 37, McN*
24	166G2-2	117-5	JRHSC	1987	Mahabaleshwar, Maharashtra	*Sr5, 7a, 7b, 8a, 8b, 13, 14, 22, 24, 25, 26, 27, 28, 30, 31, 32, 33, 34, 35, 36, 38, 39, 40, 43, Tmp/2, 6, 9a, 9b, 9c,9e, 9d, 9e, 9f, 9g, 10, 11, 12, 15, 16, 17, 19, 21, 23, 29, 37, McN*
25	37G19	117-6	KRCSC	1990	Niphad, Maharashtra	*Sr5, 8a, 8b, 9b, 22, 24, 25, 26, 27, 28, 30, 31, 32, 33, 35, 36, 37, Tmp*/*2, 6, 7a, 7b, 9e, 9f, 9g, 10, 11, 12, 13, 14, 15, 16, 17, 19, 21, 23, 29, 34, McN*
26	7G11	122	RRJQC	1952	Bagalkot, Karnataka	*Sr7a, 8a, 8b, 9e, 10, 12, 14, 15, 16, 17, 18, 19, 20, 22, 24, 25, 26, 27, 28, 30, 31, 32, 33, 35, 36, 37, 38, 39, 40, 43, Tmp, Tt3*/*5, 6, 7b, 9a, 9b, 9c, 9d, 9f, 9g, 11, 13, 21, 23, 29, 34, McN*
27	53G1	184	FPCSC	1965	Karnal, Haryana	*Sr5, 6, 9b, 15, 18, 21, 22, 24, 25, 26, 27, 28, 29, 30, 31, 32, 33, 35, 36, 37, 38, 39, 40, 42, 43, Tmp, Tt3*/*7a, 7b, 8a, 8b, 9a, 9c, 9d, 9e, 9f, 9g, 10, 11, 12, 13, 14, 16, 17, 19, 20, 23, 34, McN*
28	55G1	184-1	FPHSC	2005	Pune, Maharashtra	*Sr5, 6, 15, 18, 21, 22, 24, 25, 26, 27, 28, 29, 30, 31, 32, 33, 35, 36, 37, 38, 39, 40, 42, 43, Tmp, Tt3*/*7a, 7b, 8a, 8b, 9a, 9b, 9c, 9d, 9e, 9f, 9g, 10, 11, 12, 13, 14, 16, 17, 19, 20, 23, 34, McN*
29	7G43	295	RRHQC	1962	Indore, Madhya Pradesh	*Sr8a, 8b, 9e, 9c, 10, 12, 14, 15, 16, 18, 19, 20, 22, 24, 25, 26, 27, 28, 30, 31, 32, 33, 35, 36, 37, 38, 39, 40, 43, Tmp, Tt3*/*6, 5, 7a, 7b, 9a, 9b, 9d, 9f, 9g, 11, 13, 17, 21, 23, 29, 34, McN*

*Jin et al. (2008).

### Virulence Phenotyping

All the pathotypes were inoculated onto standard Indian stem rust differentials (Supplementary Table 1; Bahadur et al., 1985a; Nayar et al., 1997; Prasad et al., 2018) for screening their purity and virulence phenotypes. Other near isogenic lines and wheat genotypes having known Sr genes were also inoculated with all 29 *Pgt* pathotypes to derive the avirulence/virulence formula of these pathotypes. The other additional Sr genes that were used to characterize these pathotypes are mentioned against each pathotype (avirulence/virulence formula) in Table 1. The sets of differentials were grown in aluminum bread pans (29 × 12 × 7 cm) containing fresh loam soil and farmyard manure (3:1), autoclaved twice at 60°C for 2 h. Inoculation of each pathotype was performed 10–12 days after sowing, once the primary leaf of seedlings fully emerged. Approximately 50 mg uredospores were suspended in 2 ml Soltrol 170 (Chevron Phillips Chemical Company, United States), a non-phytotoxic isoparaffinic mineral oil, and this suspension was atomized on the differentials using an atomizer in the inoculation chamber. The inoculated seedlings were sprayed with a fine mist of water and then shifted to water-saturated incubation chambers with > 80% relative humidity (RH). After incubation for 48 h, the trays were transferred to the fully air-conditioned greenhouses equipped with separate growth chambers for individual pathotypes and maintained at 25 ± 2°C, 40–60% RH, and 12 h photoperiod. The infection type (IT) of each pathotype on stem rust differentials was scored 15 days after inoculation according to the 0–4 disease rating scale (Stakman et al., 1962), where ITs 0–2 were interpreted as resistant and 3–4 as susceptible. The virulence phenotyping exercise was repeated to confirm the purity of each pathotype. Likewise, the avirulence/virulence formula of each pathotype was assigned based on the ITs of each pathotype on near-isogenic lines or wheat varieties with known stem rust (Sr) resistance genes.

### Molecular Genotyping

#### DNA Extraction and Primer Synthesis

The cetyltrimethylammonium bromide (CTAB) method, as previously described (Kiran et al., 2017), was employed to extract DNA from 50 mg dried uredospores each of 29 *Pgt* pathotypes. The purity and concentration of the extracted genomic DNA were assessed using the NanoDrop 2000^®^ UV-Vis Spectrophotometer (Thermo Fisher Scientific, Waltham, MA, United States). The quality of DNA was also evaluated by running it on 1% agarose gels. One hundred *Puccinia* spp. specific SSR markers designed previously (Karaoglu et al., 2013; Prasad et al., 2017a,^2018^; Savadi et al., 2020) were synthesized from Agile Life Science Technologies (New Delhi, India) and screened against selected eleven *Pgt* pathotypes (11, 14, 15-1, 21, 24A, 34-1, 40A, 42, 117-6, 122, and 184), representing different virulence patterns. Of the 100 markers, 37 (Table 2) with the ability to amplify highly repeatable polymorphic alleles were selected to genotype all 29 pathotypes.

**TABLE 2:** List of 37 SSR markers used for population genetic diversity analysis of *Puccinia graminis* f. sp. *tritici* and their sequence, annealing temperature, major allele frequency, gene diversity, heterozygosity, and polymorphic information content (PIC) values.

S. no.	Marker/Locus	Primers (5′-3′)	AT* (°C)	MAL	GD	H	PIC	References
1	PgSUN27	F: TCAGCCCATCATCAGGACTC	54	0.53	0.5	0.86	0.6	Karaoglu et al., 2013
		R: CCTCCAGCCCAGTTCAGAGC						
2	PGTG 03066	F: CGAAAGAAAGGAAACGAAGGT	50	0.53	0.5	0.66	0.88	Prasad et al., 2018
		R: ACATCAATCTCGACCAATCTCC						
3	PGTG 04483	F: TCCCATCACATGCAGTAGTAGC	48	0.5	0.5	0.93	0.69	
		R: AATCTAATTGACAGCCTTGCGT						
4	PGTG 00856	F: ACAACAACAACAGCAGGACATC	49	0.6	0.48	0.45	0.79	
		R: TCGTTGAGGATGATTGAGTTTG						
5	PGTG 07438	F: ACTGGCTCATCATCATCTTCCT	52	0.55	0.49	0.9	0.49	
		R: CCAACCATTCCGACCTAATAAA						
6	SSR-P GT-42	F: GGGGTGAGTTTCTGTATTGA	50	0.66	0.45	0.55	0.91	Prasad et al., 2017a
		R: CAGAGATCATCGAGGAAAAC						
7	SSR-P AG-40	F: CTTTCTTACCCCCACAACTAC	51	0.67	0.44	0.52	0.87	
		R: CTCTCTCTCTCTCTCTCTCTCTC						
8	SSR-P CT-36	F: ACTCTCAAACTCACTCCCTCT	48	0.74	0.38	0.17	0.83	
		R: GACTACACCATTTCAAACCAA						
9	SSR-P AC-32	F: ACAAAACAAACAGATCCACTG	49	0.53	0.5	0.17	0.91	
		R: ACGTATTTGGTCTTCTTCTCC						
10	SSR-P CAA-60	F: AACTGCGAGGACAACTTTC	52	0.81	0.31	0.38	0.97	
		R: CGTCTGCTGAGTTTCTGTATT						
11	SSR-P GGT-45	F: GCTGCTTGATGGAGGATG	55	0.55	0.49	0.62	0.75	
		R: AACAGCTTCAGCGACCTC						
12	SSR-P GTT-45	F: GATGAGGTTGTTGAAGGAGA	49.6	0.52	0.5	0.76	0.81	
		R: ACCAGAACCAACAAAACAAC						
13	SSR-P CAC-45	F: GAAGACCATCCTCACGACT	51	0.66	0.45	0.14	0.69	
		R: TTCTTCTTGTTGGTTTTTCTG						
14	SSR-P CAAC- 44	F: AGCGTAGAGTCAGTCAGTCAG	51	0.59	0.49	0.41	0.61	
		R: GCTAATAAGGAGATTGGGTTG						
15	SSR-P TATC-40	F: AAGCGTGATCAAGTAGGTTTA	50.4	0.74	0.38	0.38	0.47	
		R: GATGGACAAGTAGAGAGATGG						
16	SSR-P TCCG-36	F: TTTTTCTAGATCCACCAACC	50.4	0.52	0.5	0.48	0.71	
		R: TACGAACAGGAGTCCCTCA						
17	SSR-P TATTG-60	F: TCAAACAACTTCATCCTGAAC	48	0.62	0.47	0.28	0.87	
		R: ATGTGATATCTTTTGGATTGG						
18	SSR-P TCTTT-50	F: GGGTTTATATGGTGGGTGT	48	0.53	0.5	0.52	0.78	
		R: GTTGAGTGGGTGAGATGAGTA						
19	SSR-P ACAAAC-48	F: ATACATTTTGGTTACCCACCT	48.9	0.57	0.49	0.66	0.8	
		R: TGTGTTTGTTTGTGTTTGTGT						
20	SSR-P GCTGTT-60	F: GATGAGCAGCATGAGGAG	51.9	0.53	0.5	0.38	0.81	
		R: CACCAGAACAACATACTCCAT						
21	PtESSR6	F: ATGATGTCCCGCTCACCT	52	0.69	0.43	0.28	0.88	Savadi et al., 2020
		R: ATCACAGAGTTGGCGATATG						
22	PtESSR17	F: CAAACTGCCCAATCTTTATCT	53	0.53	0.5	0.31	0.77	
		R: GTGCGAGCCTGTCCCTTC						
23	PtESSR18	F: CTCTGCCCCTCTCTCTCC	50	0.57	0.49	0.52	0.76	
		R: CTACCTCATCAGGCACCTT						
24	PtESSR22	F: ACAGAGGGAGCTCCACAA	51	0.69	0.43	0.62	0.79	
		R: CTCCCGCTACCCTTTCTC						
25	PtESSR24	F: CGTAGACGTTCACCTCGT	49	0.76	0.37	0.41	0.58	
		R: GGCGGTTACTGTTTTGTTT						
26	PtESSR25	F: TCTCGACGATCTGGACAT	50	0.6	0.48	0.66	0.49	
		R: GAGGTCGAGGACGAGGAC						
27	PtESSR26	F: AGGGAGGAGGATGATGGT	55	0.69	0.43	0.48	0.76	
		R: TGGAGGAGAAAGGATGAAC						
28	PtESSR27	F: GGATGAGAGATACAACAACCA	53	0.59	0.49	0.48	0.82	
		R: AACATTTGGGTGCAGTAAATA						
29	PtESSR28	F: ATTGTGGCGGCGGAGGAG	54	0.97	0.07	0.07	0.61	
		R: GATCTTGGACACCGAGAAG						
30	PtESSR30	F: GGACTTGCGTTCTACTACAAA	53	0.93	0.13	0.14	0.48	
		R: TACTCCACTTTTTAGCCTCCT						
31	PtESSR31	F: TCTCGAGGATCTCTAGGTAGC	53	0.55	0.49	0.41	0.86	
		R: GACGAGACCTCCGTATCC						
32	PtESSR33	F: AGTGACACCATGAATGAAAAA	53	0.79	0.33	0.21	0.7	
		R: CAAGAAAACAAAAACAGCACT						
33	PtESSR34	F: CATATGAAGACAGGGAGCAC	54	0.81	0.31	0.31	0.43	
		R: GTCATGGTGGATTGATTGA						
34	PtESSR35	F: GATTCCGGATTAGCCACTA	53	0.66	0.45	0.48	0.62	
		R: AAATAAGCAGCTCCCAATC						
35	PtESSR36	F: CTGTTTCTTGGTGATCAGGT	54	0.78	0.35	0.31	0.62	
		R: CCAGAACAGTCATCCTCCT						
36	PtESSR38	F: CTTGCTGTGCCGGTCCTT	53	0.64	0.46	0.52	0.76	
		R: CCTCTCCACCACCATGAC						
37	PtESSR46	F: TCCCAGAGTATGTGTTTTGTT	53	0.74	0.38	0.38	0.67	
		R: CGTGAGTTATGGATGGATG						
Mean				0.65	0.43	0.45	0.73	

*AT, annealing temperature; MAL, major allele frequency; GD, gene diversity; H, heterozygosity; PIC, polymorphic information content.

#### Polymerase Chain Reaction Amplification and Polymorphic Allele Scoring

Polymerase chain reaction (PCR) was performed in 20 μl of the PCR reaction mixture containing 25 ng of template DNA, 200 μM of dNTPs, 1 × PCR buffer (10 mM Tris, pH 9.0, and 50 mM KCl), 1.5 mM MgCl_2_, 2.5 U Taq polymerase (HiMedia Lab., Mumbai, India), and 10 pmol of both forward and reverse primers. The PCR programs were set at 94°C for 2 min (initial denaturation); followed by 35 cycles of 30 s at 94°C, 30 s at marker specific annealing temperature (Table 2), and 1 min at 72°C; with a final extension at 72°C for 7 min in a thermal cycler (Applied Biosystems Veriti™, CA, United States). The amplified products were resolved on 3% Super MT4 Agarose gel (Life Technologies, New Delhi, India) in 1× Tris-Borate-EDTA (TBE) buffer at 65–70 V for 2–3 h. DNA fragments were visualized under UV light and photographed using the gel documentation system (Bio-Rad Laboratories Inc., Hercules, CA, United States). The number of polymorphic alleles for each SSR locus was scored. The genotyping studies were repeated and confirmed before data analysis.

### Data Analysis

The virulence frequency (VF) of all 29 pathotypes on each of the entry (gene/cultivar) of differential sets was determined as the percentage of the pathotype virulent for a specific gene or cultivar of the differential set from the total of pathotypes under study. The binary data, generated as “1” and “0” to symbolize the virulence and avirulence, respectively, of each pathotype on wheat differential lines, were used to construct the virulence phenotype-based dendrogram of *Pgt* pathotypes. Likewise, binary codes as “1” and “0” were generated to represent the presence and absence, respectively, of SSR alleles of different sizes from multilocus allelic data matrix for all pathotype–primer combinations. The SSR alleles of different sizes from multilocus allelic data matrix were arranged according to their molecular weights (in base pair) for calculating major allele frequency (MAF), gene diversity, and heterozygosity of each SSR marker using PowerMarker version 3.25 (Lu et al., 2005). Polymorphic information content (PIC) value, discriminating two loci, was calculated using the following formula proposed by Anderson et al. (1993):


(1) $$\text{PICj}=1-\sum_{f=1}^{n}\left(P_{ij}\right)2$$

where *n* and *P*_ij_ are the numbers of alleles and frequency of the *j*th allele for marker *I*, respectively.

For the analysis of genetic diversity parameters using GenAlEx, the *Pgt* population was divided into the following five subpopulations: (i) North India, (ii) Karnataka, (iii) Madhya Pradesh, (iv) Maharashtra, and (v) Tamil Nadu, which correspond to the place of origin of different pathotypes. The Shannon’s information index (*I*), expected heterozygosity (He), unbiased expected heterozygosity (uHe), percentage of polymorphic loci (%P), principal coordinate analysis (PCoA), Mantel correlation test, and analysis of molecular variance (AMOVA) were determined using GenAlEx 6.5 (Peakall and Smouse, 2012). GenAlEx was also used to calculate Nei’s genetic distance in a pairwise population matrix. Cluster analysis for both virulence phenotype and molecular genotype data was performed to generate dendrograms based on the neighbor-joining (NJ) cluster analysis using DARwin 6.0.14 (Perrier and Jacquemoud-collet, 2006). The population structure data of *Pgt* pathotypes were generated using STRUCTURE 2.3.4 (Pritchard et al., 2000) with the admixture model-based clustering method with 50,000 burin-in periods, 500,000 iterations, and two replications.

## Results

### Virulence Phenotyping and Frequency

**FIGURE 2:**
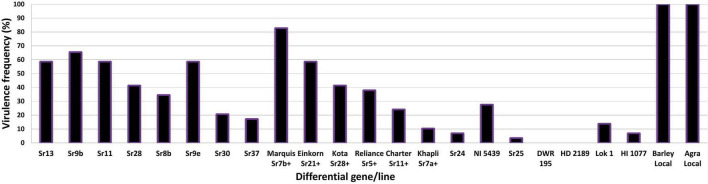
Virulence frequencies (%) of *P. graminis* f. sp. *tritici* pathotypes on stem rust differentials.

**FIGURE 3:**
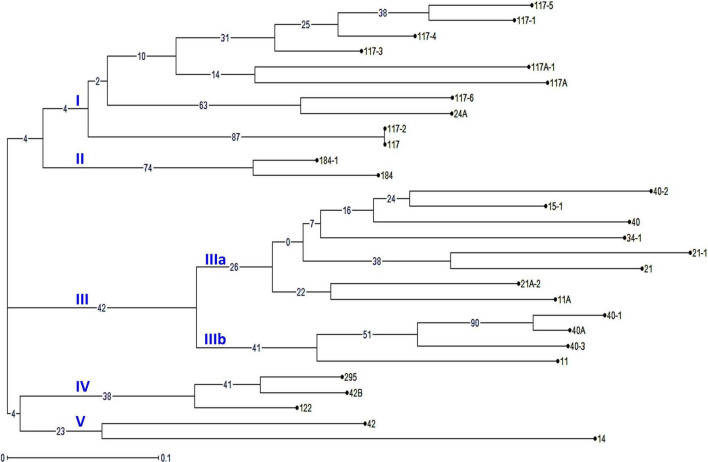
The phylogenetic tree of *P. graminis* f. sp. *tritici* pathotypes from the Indian subcontinent based on their avirulence/virulence phenotypes on stem rust differentials. The characters at the base of selected branches indicate the number of the virulence group and subgroups.

The virulence phenotyping of 29 *Pgt* pathotypes detected over the 90 years was performed on Indian stem rust differential sets and other Sr genes to explore their avirulence/virulence formula. Avirulence/virulence formula established for each of the 29 *Pgt* pathotypes against up to 50 Sr genes is presented in Table 1. Ten Sr genes, namely, *Sr26, Sr27, Sr31, Sr32, Sr33, Sr35, Sr39, Sr40, Sr43, SrTmp*, and *SrTt3* were found effective against all 29 pathotypes (Table 1). The *Sr24* was effective against all the pathotypes except 34-1 and 40-1, likewise, *Sr25* conferred resistance to all the pathotypes except 40-2. The virulence phenotyping observations were used to calculate the VF of all 29 pathotypes on each of the entry of differential sets. The VF of *Pgt* pathotypes on stem rust differentials varied from 0.00 {DWR195 (*Sr31*) and HD2189 (*Sr11* and some other unknown gene)} to 100% (for Barley Local and Agra Local) (Figure 2). The VF on Khapli (*Sr7a, Sr13, Sr14*+), *Sr7a* +, *Sr24*, HI1077 (*Sr9b, 11*+), and *Sr25* was less than 10.4%, whereas > 40% VF was recorded on *Sr13*, *Sr9b*, *Sr11*, *Sr9e*, Marquis (*Sr7b*+), Einkorn (*Sr21*+), and Kota (*Sr28*+). Moderate virulence frequencies was observed on Lok 1 (*Sr9b, 11*+) (13.8%), *Sr37* (17.24%), *Sr30* (20.68%), Charter (*Sr11*+) (24.13%), NI 5439 (*Sr11*+) (27.58%), *Sr8b* (34.84%), and Reliance (*Sr5*+) (37.9%). The dendrogram generated by unweighted NJ cluster analysis using virulence phenotype data of each pathotype on all the differentials divided the *Pgt* pathotypes into five distinct virulence groups (VGs) (I–V) with 10, 2, 12, 3, and 2 pathotypes, respectively, in each group (Figure 3). Group III was further divided into two subgroups IIIa and IIIb with eight and four pathotypes in each, respectively. All nine pathotypes in race 117 lineage were positioned in group I along with 24A, which to a certain extent have similar virulence pattern as 117-6. Only two pathotypes (184 and 184-1) were clustered in VG II. Pathotypes belonging to race 40 lineage were placed in VG III along with other pathotypes, i.e., race 21 group (21, 21-1, and 21A-2), race 11 group (11 and 11A), 15, and 34-1. Pathotypes 40A, 40-1, and 40-3 with a similar avirulence/virulence formula were clustered in subgroup IIIb (Figure 3).

### Possible Evolutionary Hierarchy of Indian *Puccinia graminis* f. sp. *tritici* Pathotypes

**FIGURE 4:**
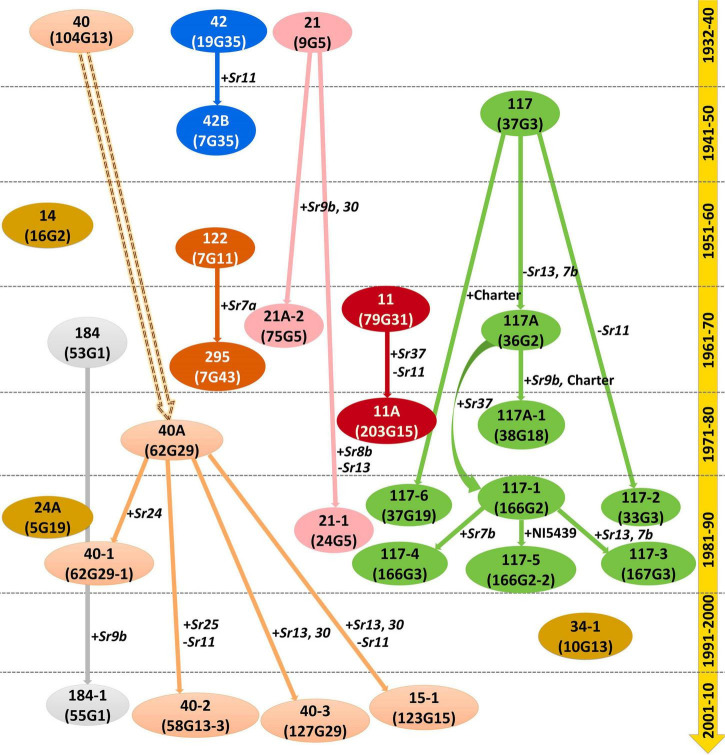
Proposed virulence-based hypothetical evolutionary hierarchy of *P. graminis* f. sp. *tritici* pathotypes from the Indian subcontinent based on virulence phenotyping. Solid outlines explain the probability of mutations for gain or loss of virulence for the Sr genes indicated alongside. The parallel dotted lines indicated the timeline of pathotype evolution in decades.

Based on the avirulence/virulence assay on the standard differentials possessing different Sr genes, we proposed a possible evolutionary hierarchy among 29 *Pgt* pathotypes collected from India and neighboring countries over the nine decades (Figure 4). Seven different possible lineages have been projected to explain the evolutionary pattern of these pathotypes over the years: (i) race 11 (2 pathotypes), (ii) race 21 (3 pathotypes), (iii) race 40 (6 pathotypes), (iv) race 42 (2 pathotypes), (v) race 117 (9 pathotypes), (vi) race 122 (2 pathotypes), and (vii) race 184 (2 pathotypes). The remaining three pathotypes, namely, 14, 24A, and 34-1 first discovered in 1959, 1981, and 1991, respectively, may be incursions from elsewhere or emerged independently. No conclusive evidence is available to prove the existence of their variants in the Indian subcontinent.

### Molecular Genotyping

#### Simple Sequence Repeat Loci

Out of 100 *Puccinia* spp. SSR markers screened against *Pgt* pathotypes, 37 were found polymorphic. From 29 *Pgt* pathotypes used in this study, a total of 175 alleles with 4.7 alleles per marker were amplified. The number of alleles per locus ranged from two to eight. The amplification product sizes of these markers ranged from 0.1 to 1.5 kb (Supplementary Figure 1 and Supplementary Table 2). The MAF of polymorphic markers ranged between 0.50 (PGTG 04483) and 0.97 (PtESSR28), with three other markers (i.e., SSR-P CAA-60, PtESSR30, and PtESSR34) showing ≥ 0.80 MAF (Table 2). Gene diversity ranged from 0.13 (PtESSR30) to 0.50 (Nine markers). The heterozygosity of these markers was in the range of 0.14 (SSR-P CAC-45 and PtESSR30) to 0.97 (PGTG 04483). Three polymorphic markers (PgSUN27, PGTG 04483, and PGTG 07438) had a heterozygosity score ≥0.80. The PIC value ranged from 0.43 (PtESSR34) to 0.97 (SSR-P CAA-60) with thirteen markers showing ≥ 0.80 PIC value (Table 2).

### Molecular Grouping

#### Phylogenetic Analysis and Principal Coordinate Analysis

**FIGURE 5:**
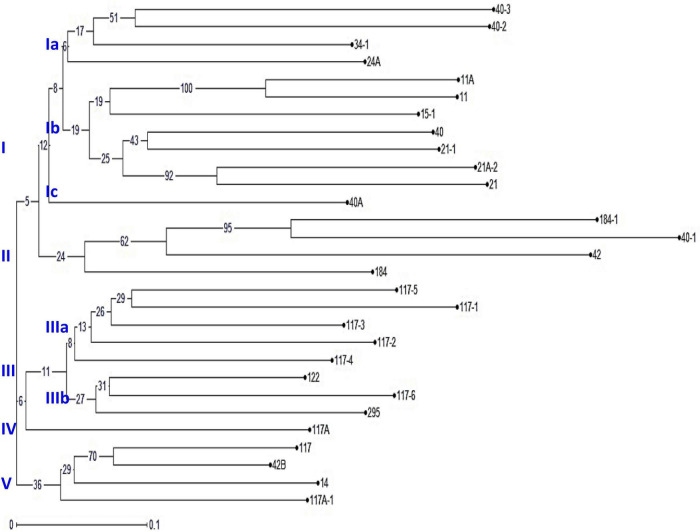
The phylogenetic tree of *P. graminis* f. sp. *tritici* pathotypes from the Indian subcontinent constructed using 37 polymorphic simple sequence repeat (SSR) markers. The characters at the base of selected branches indicate the number of the molecular group and subgroups.

**FIGURE 6:**
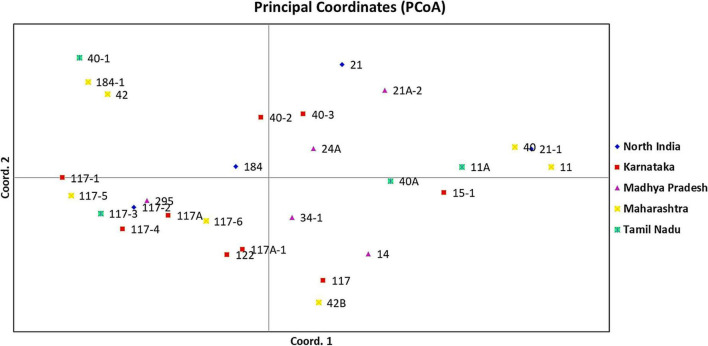
Principal coordinate analysis (PCoA) of *P. graminis* f. sp. *tritici* pathotypes from different geographical regions of India using 37 polymorphic SSR markers. The first and second principal axes revealed 13.03% and 12.08% of the total variation, respectively.

An unrooted dendrogram generated by unweighted NJ cluster analysis of the molecular data generated five distinct molecular groups (MGs I–V) with 12, 4, 8, 1, and 4 pathotypes, respectively, in each group. MGs I and III were further divided into three (i.e., Ia, Ib, and Ic) and two (i.e., IIIa and IIIb) subgroups, respectively (Figure 5). Subgroup Ia had four pathotypes (i.e., 24A, 34-1, 40-2, and 40-3), while Ib had seven pathotypes of which pathotypes 11 and 11A, belonging to race 11 lineage based on their virulence, had identical SSR genotype supported by a bootstrap value of 100%. Likewise, pathotypes 21 and 21A-2, belonging to the race 21 lineage and placed in subgroup Ib, shared 92% similarity in SSR genotype as supported by the bootstrap value. MG II had four pathotypes, namely, 40-1, 42, 184, and 184-1, the latter two of which had 95% similar SSR genotype as indicated by the bootstrap value. The third MG had five pathotypes in subgroup IIIa, all belonging to race 117 lineage, while subgroup IIIb had three pathotypes 117-6, 122, and 295. MG IV and subgroup Ic comprised only a single pathotype, i.e., 117A and 40A, respectively, demonstrating poor genetic similarity of these pathotypes with others. The MG V comprised of four pathotypes, namely 14, 117A-1, 42B, and 117, the latter two had fairly similar SSR genotype with 70% bootstrap value. The scatter plot from the PCoA revealed that the first two axes contributed 25.11% cumulative variance with 13.03% and 12.08% variation explained by the first and second axes, respectively (Figure 6 and Supplementary Table 3). The cumulative variance interpreted by all three axes together was 33.11%. However, this analysis, like virulence-based clustering, could not justify the region-specific grouping of the *Pgt* pathotypes.

#### Population Diversity

The genetic diversity of different *Pgt* populations was assessed using several parameters including the number of observed (Na) and effective (Ne) alleles, Shannon’s information index (I), expected heterozygosity (He), and percentage of polymorphic loci (%P) (Table 3). Comparing the different regions, Karnataka and Maharashtra had the highest Na (1.64), followed by Tamil Nadu (1.48), Madhya Pradesh, and North India (both 1.46). The Ne ranged between 1.39 (North India) and 1.47 (Maharashtra). The Ne for other regions, i.e., Karnataka, Madhya Pradesh, and Tamil Nadu was 1.46, 1.43, and 1.43, respectively. The highest *I*-value was observed for Maharashtra (0.41) and the lowest for North India (0.35). Maharashtra and Karnataka had the highest (both 0.27) while North India (0.23) had the lowest He. Equal He (0.25) was observed for Madhya Pradesh and Tamil Nadu. The highest uHe score was 0.29 for both Maharashtra and Tamil Nadu, while lowest for North India (0.26). The %P ranged from 65.71 (North India) to 79.43 (Maharashtra). It was 66.29, 70.86, and 78.29 for Madhya Pradesh, Tamil Nadu, and Karnataka, respectively (Table 3). The Nei’s genetic distance in pairwise population matrix was very low for all the geographical combinations, and it ranged from 0.041 between Tamil Nadu and Maharashtra to 0.087 between Karnataka and North India (Table 4).

**TABLE 3:** Values of different genetic diversity parameters (number of observed and effective alleles, Shannon information index, expected and unbiased expected heterozygosity, and percentage of polymorphic loci) of *Puccinia graminis* f. sp. *tritici* groups belonging to five different geographic regions.

Population	Population size	Na ± SD	Ne ± SD	I ± SD	He ± SD	uHe ± SD	%P
North India	4	1.46 ± 0.06	1.39 ± 0.02	0.35 ± 0.02	0.23 ± 0.01	0.26 ± 0.01	65.71%
Karnataka	9	1.64 ± 0.05	1.46 ± 0.02	0.40 ± 0.02	0.27 ± 0.01	0.28 ± 0.01	78.29%
Madhya Pradesh	5	1.46 ± 0.06	1.43 ± 0.02	0.37 ± 0.02	0.25 ± 0.01	0.27 ± 0.01	66.29%
Maharashtra	7	1.64 ± 0.05	1.47 ± 0.02	0.41 ± 0.01	0.27 ± 0.01	0.29 ± 0.01	79.43%
Tamil Nadu	4	1.48 ± 0.06	1.43 ± 0.02	0.38 ± 0.02	0.25 ± 0.01	0.29 ± 0.01	70.86%

*Na, no. of observed alleles; Ne, no. of effective alleles; I, Shannon’s information index; He, expected heterozygosity; uHe, unbiased expected heterozygosity; %P, percentage of polymorphic loci.*

**TABLE 4:** Pairwise population matrix of Nei’s genetic distance.

	North India	Karnataka	Madhya Pradesh	Maharashtra	Tamil Nadu
North India	0.000				
Karnataka	0.087	0.000			
Madhya Pradesh	0.086	0.070	0.000		
Maharashtra	0.083	0.063	0.074	0.000	
Tamil Nadu	0.080	0.070	0.080	0.041	0.000

#### Analysis of Molecular Variance

The AMOVA carried out by segregating the total variation among and within the populations explained that the maximum genetic variation was within the populations rather than among the *Pgt* populations belonging to different geographic regions. Genetic variations within the populations were 92%, and the remaining 8% (at *P* = 0.001) genetic variations were among the *Pgt* populations belonging to different geographic regions (Supplementary Figure 2 and Supplementary Table 4).

#### Population Structure

**FIGURE 7:**
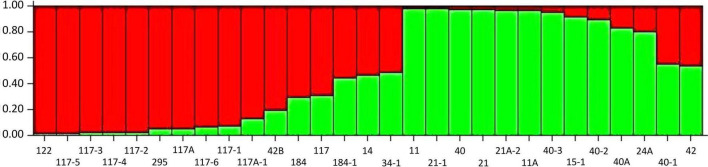
Population grouping of *P. graminis* f. sp. *tritici* pathotypes at estimated membership fraction for *K* = 2 using STRUCTURE. The grouping indicates two different populations in green and red with allele sharing more than 70% and an admixture with allele sharing less than 70%.

The SSR marker data-based population structure analysis was carried out to understand the genetic association among 29 *Pgt* pathotypes using STRUCTURE version 2.3.4. The maximum likelihood values and the mode of the distribution of the Δ*K* index were 2, indicating the existence of two subpopulations. However, seven pathotypes (184, 117, 184-1, 34-1, 14, 40-1, and 42) were designated as admixture individuals and anticipated to have mixed ancestry. The *K* = 2 divided all the pathotypes into two subpopulations (S1 and S2) consisting of 11 *Pgt* pathotypes in each that were originated in 5 different geographical regions (Figure 7 and Supplementary Table 5). However, we did not observe any geographical region-specific grouping of *Pgt* pathotypes in both the subpopulations, signifying that the geographical distances have very less influence on genetic variations in *Pgt* populations.

#### Mantel Correlation Test

Correlation analysis was performed to examine the relationship between virulence phenotypes and SSR genotypes of the *Pgt* population consisting of 29 individuals using the Mantel correlation test. The analysis revealed that there was a positive but weak (*R*^2^ = 0.1546) correlation between these two variables. The linear regression equation of correlation analysis was *y* = 0.0843*x* + 1.8141 (Supplementary Figure 3).

## Discussion

*Puccinia graminis tritici* (*Pgt*), causing wheat stem rust, is highly destructive and one of the most extensively studied plant pathogens. Stem rust is a serious threat to world wheat production and food security. The emergence and dispersal of *Pgt* races, such as TTKSK (Ug99), TTRTF, TKTTF (Digalu race), and their variants, have posed a serious risk to global wheat production (Pretorius et al., 2000; Olivera et al., 2015; Patpour et al., 2020). The occurrence of such virulence has not been reported from any wheat-growing regions in the Indian subcontinent; however, strategic monitoring to track the change in the virulence pattern of *Pgt* is in place for early detection of likely new virulence (s), which might be introduced from elsewhere or evolved locally.

This study reports virulence phenotype and SSR genotype-based diversity analysis of *Pgt* repository (29 pathotypes) from the Indian subcontinent. Virulence phenotype data revealed both inter- and intra-lineage diverse nature of all 29 pathotypes on stem rust differentials and other Sr genes (Table 1). The virulence frequencies of *Pgt* pathotypes on stem rust differentials ranged between 0% and 100%. It was in the range of 20%–60% for the majority of the differentials. Ten Sr genes (i.e., *Sr26, Sr27, Sr31, Sr32, Sr33, Sr39, Sr40, Sr43, SrTmp*, and *SrTt3*) were found effective to the Indian population of *Pgt*, whereas virulence for *Sr24* and *Sr25* was found only in two (34-1 and 40-1) and one pathotypes (40-2), respectively. Among the effective all stage resistance (ASR) genes in India, seven (*Sr26, Sr27, Sr32, Sr33, Sr39, Sr40*, and *Sr43*) confer resistance to Ug99 and other variants in its lineage and are possibly the most useful ASR genes (Singh et al., 2015). Some of these genes, *Sr26* in particular, have performed well against stem rust pathotypes worldwide and, therefore, could be a valuable asset for utilization in breeding programs if used in combinations with other effective genes (Singh et al., 2015; Terefe et al., 2016). Moreover, the availability of promising slow-rusting adult plant resistance (APR) genes such as *Sr2*, *Sr55*, *Sr56*, *Sr57*, *Sr58*, and others, and their pyramiding with effective ASR genes in cultivars with superior agronomic traits could further improve the effectiveness and durability of stem rust resistance (Huerta-Espino et al., 2020).

Virulence phenotype-based cluster analysis grouped *Pgt* pathotypes into five distinct VGs (I–V). All the pathotypes in race 117 lineage were in VG I (Figure 3). The clustering of pathotypes 40A, 40-1, and 40-3 justifies their similar virulence phenotypes on majority of the differentials except for: (i) *Sr24* (virulent: 40-1; avirulent: 40A and 40-3), (ii), *Sr13*, and (iii) *Sr30* (virulent: 40-3; avirulent: 40A and 40-1; for both *Sr13* and *Sr30*). The identical virulence phenotypes of 40A and 40-1, except for *Sr24* (virulent: 40-1), suggested and have been established that pathotype 40-1 had evolved through a single-step gain of virulence for *Sr24* in 40A (Bhardwaj et al., 1990). Other pathotypes in the same race lineage and belonging to one VG include 184 and 181-1 in VG II, 122 and 295 in VG IV, and 21, 21A-2, and 21-1 in subgroup IIIa. Likewise, pathotypes 11 and 11A were clustered in subgroups IIIa and IIIb (VG III), respectively.

The possible evolutionary lineages deduced based on the virulence-avirulence assays of 29 *Pgt* pathotypes classified them into seven lineages. In race 11 lineage, the first pathotype was 11 (virulence for *Sr11* and *Sr30*), which was detected in 1962 from Maharashtra (India). Subsequently, another pathotype in its lineage with additional virulence on *Sr37* was identified in 1974 as 11A (Sinha et al., 1976). Other difference between pathotypes 11 and 11A is that the latter has an avirulence phenotype on *Sr11*. In race 21 lineage, the first pathotypes to be identified was 21, which was simultaneously detected from Lyallpur, Pakistan, and Himachal Pradesh (India) in 1935. The one-step gain in virulence for *Sr9b* and *Sr30* in pathotype 21 caused the emergence of 21A-2 in 1962 from Madhya Pradesh (India). Likewise, additional virulence for *Sr8b* and loss of virulence for *Sr13* in pathotype 21 resulted in the emergence of 21-1 in 1985 from Uttar Pradesh (India) (Bahadur et al., 1985b). Pathotype 40 in race 40 lineage was detected in 1932 from Pune (India; Mehta, 1940), while a new independent pathotype 40A emerged in the Nilgiri Hills (Wellington, India) in 1974 (Sharma et al., 1975). Additional virulence for *Sr24* in pathotype 40A resulted in the emergence of 40-1 (Bhardwaj et al., 1990), while simultaneous gain and loss of virulence for *Sr25* and *Sr11*, respectively, in 40A resulted in the emergence of 40-2 (Jain et al., 2009). Other pathotypes 40-3 and 15-1 in race 40 lineage emerged from Dharwad (Karnataka) in 2008, resulting from the gain of virulence for *Sr13* and *Sr30* (for both 15-1 and 40-3) and loss of virulence for *Sr11* (for 15-1) in 40A (Jain et al., 2013).

Race 117 lineage has been the most variable collection of *Pgt* pathotypes in the Indian subcontinent. Pathotype 117 in this lineage was first detected from Madhya Pradesh in 1945 (Prasada and Lele, 1952). Since then, eight additional variants of this pathotype have been detected. The loss of virulence for *Sr7b* and *Sr13* in pathotype 117 resulted in the emergence of 117A in 1960 from Karnataka (Patil et al., 1963). Consequently, pathotypes 117-2 and 117-6 evolved as a result of loss of virulence to *Sr11* (Bhardwaj et al., 1990) and one step gain of virulence on Charter (Bhardwaj et al., 1994), respectively, in pathotype 117. Additional virulence in pathotype 117A for *Sr9b* and Charter resulted in the emergence of 117A-1 (Sharma et al., 1979), while the gain in virulence for *Sr37* in 117A contributed to the advent of 117-1 (Bhardwaj et al., 1989). Added virulence for *Sr7b* and NI5439 in pathotype 117-1 resulted in the emergence of 117-4 and 117-5, respectively, whereas pathotype 117-1 advanced to 117-3 after gaining additional virulence for *Sr7b* and *Sr13* (Bhardwaj et al., 1994). The gain in virulence was the principal mode of evolution in the race 117 lineage except for the emergence of 117A (117-*Sr13* and *Sr7b*) and 117-2 (117-*Sr11*), where the loss of virulence in pathotype 117 resulted in their evolution. The virulence-based hypothetical evolutionary hierarchy proposed in this study is to a great extent in agreement with previously discussed virulence phenotype data-based NJ cluster analysis. The evolutionary behavior of *Pgt* isolates reveals that the gain of virulence for *Sr13* was most frequent. The resistance genes present in the most widely used cultivars always play a critical role in influencing the prevalence of pathogen races in a specific region or location. The *Sr13*, one of the most widely used Sr genes in durum wheat, is very common in Indian cultivars as revealed by gene postulation using the gene matching technique. Moreover, the areas covering Madhya Pradesh, Karnataka, Maharashtra, and Tamil Nadu states, where most of the new pathotypes were initially detected, have all three types of wheat (i.e., aestivum, durum, and dicoccum) in cultivation. Adequate host diversity among aestivum, durum, and dicoccum wheat cultivars might be facilitating easier survival for newly evolved rust isolates.

The SSR genotype pattern of each of 29 *Pgt* pathotypes was estimated using 37 *Puccinia* spp. specific SSR markers developed in previous studies (Karaoglu et al., 2013; Prasad et al., 2017a, 2018; Savadi et al., 2020). Four of these markers (i.e., PGTG 03066, PGTG 04483, PGTG 00856, and PGTG 07438) were gene-based functional SSRs, which are known to be most reliable and reproducible for genetic diversity analysis (Anderson and Lubberstedt, 2003; Zhong et al., 2009). The MAF, gene diversity, heterozygosity, and PIC values indicated that the SSR markers used in this study were highly informative and, therefore, yielded good quality genetic diversity analysis data. Thirty-two SSRs had PIC values more than 0.50, which is considered the most appropriate for conducting genetic diversity analysis (Botstein et al., 1980; Szabo, 2007).

The cluster analysis based on the SSR genotype data grouped 29 *Pgt* pathotypes into five MGs, of which MGs I and III were further divided into three and two subgroups, respectively (Figure 5). The results of the PCoA explaining 25.11% cumulative variance corroborated the NJ cluster analysis. The *Pgt* pathotype grouping as explained through NJ cluster analysis and PCoA was entirely independent of their geographical distribution. For instance, the pathotypes from different regions, i.e., 117/117A-1 (Karnataka), 42B (Maharashtra), and 14 (Madhya Pradesh) were grouped in cluster MG-V, which reflected high genetic similarity among them (Figure 5). Similarly, pathotypes 21, 21-1, and 21A-2 shared almost analogous virulence phenotype and SSR genotypes but detected in distal geographical regions, i.e., 21 and 21-1 in North India and 21A-2 in Central India. This relationship of the geographical distribution of *Pgt* pathotypes with their SSR genotypes could also be endorsed by the findings of AMOVA (Supplementary Figure 2 and Supplementary Table 4). An explanation to justify such distribution of *Pgt* pathotypes could be the existence of gene flow (the transfer of genetic information from one population to the other) over long distances. Similar findings for cereal rust pathogens are reported from India, South America, North America, and Europe (McCallum et al., 1999; Ordonez and Kolmer, 2007; Prasad et al., 2017a). Conversely, clonal lineages were described for *Puccinia* spp. evolution from Australia {*Pgt* (Zhang et al., 2017), *P. coronata* f. sp. *avenae* (Brake et al., 2001), and *P. graminis* f. sp. *avenae* (Keiper et al., 2006)} and South Africa {*Pgt* (Visser et al., 2009)}.

The correlation analysis of virulence phenotype and SSR genotype data of all *Pgt* pathotypes revealed a positive (*R*^2^ = 0.1546) correlation between these two variables, which suggests a poor correlation between the virulence phenotypes and SSR genotypes. This is logical because the SSRs used in this study were unrelated to pathogenicity/virulence-specific genes. Similar findings were observed in our earlier studies (Prasad et al., 2017a,^2018^; Gangwar et al., 2021). Such correlation was also witnessed in the *Puccinia striiformis* population by Chen et al. (2010), who clarified that SSR genotypes were independent of virulence and that the whole genomic region of the pathogen evolved much faster than its pathogenicity genes. In contrast, a strong correlation between virulence phenotype and SSR genotype data was observed in a population of *Puccinia triticina* from China (Kolmer, 2015) and Canada (Wang et al., 2010) and of *P. striiformis* from Europe (Hovmoller et al., 2002) populations. Such a positive strong correlation could be the result of SSR genotypic data acquired by the markers designed from virulence/pathogenicity genes, which are distributed throughout the pathogen genome (Ordonez and Kolmer, 2009; Kolmer, 2015). Moderate correlation (0.43; *P* = 1.0) between virulence phenotypes and SSR genotypes was reported for the *P. triticina* population from North America (Ordonez and Kolmer, 2009).

The value of different genetic diversity parameters, namely, Na, Ne, I, He, uHe, and %P was maximum for the *Pgt* population from Maharashtra and minimum for North India. These results suggest that the *Pgt* population is highly diverse in Maharashtra followed by Tamil Nadu, while the North Indian population has poor genetic diversity. These findings support our previous results for *P. triticina* and *Pgt* populations in the Indian subcontinent, wherein we documented that wheat cultivation round the year and availability of green bridges in higher hills of Tamil Nadu (Nilgiris), which receives high UV intensity sunlight, might be helping these pathogens to mutate and survive without difficulty (Prasad et al., 2017a,^2018^). All these factors are known to be critical for the evolution and survival of new virulences in nature (Cheng et al., 2014; Singh et al., 2015; Zhao et al., 2020). The new *Pgt* isolates evolved in these hills migrate to the foothills of Tamil Nadu and Karnataka, and subsequently, to plains of peninsular India (Karnataka, Maharashtra) and Central India (Madhya Pradesh), where these are established on susceptible wheat cultivars (Mehta, 1952). There are speculations that the primary source of inoculum of *Pgt* is also present in parts of Karnataka other than Nilgiris (Bhardwaj et al., 1989). Furthermore, the cultivation of susceptible bread, durum, and diacoccum wheat species in these areas might be helping an array of *Pgt* pathotypes with a diverse avirulence/virulence formula to survive and evolve locally. In contrast, cultivation of genetically uniform wheat species (mostly aestivum) in a small stem rust-prone geographical area in North India together with a short favorable period for stem rust development followed by harsh summers prohibits *Pgt* isolates to evolve and survive until the next crop season, which is reflected by poor genetic diversity within *Pgt* population in this region (Bhardwaj et al., 2019). The SSR genotype-based population structure analysis using a model-based tool clustered 29 *Pgt* pathotypes into two major subpopulations (i.e., SP1 and SP2), containing eleven pathotypes in each, and an admixture comprising seven pathotypes (i.e., 184, 117, 184-1, 34-1, 14, 40-1, and 42). Similar differentiation comprising subpopulations and admixture, using population structure analysis, was reported for the *Pgt* population from South Africa (Terefe et al., 2016; Boshoff et al., 2018) and *P. graminis* f. sp. *avenae* population from Australia, Brazil, and Sweden (Gnocato et al., 2018). However, four subpopulations were explained by a combined analysis of *Pgt* isolates from South Africa and Australia (Visser et al., 2019). Like cluster analysis, PCoA, and AMOVA, the grouping of *Pgt* pathotypes explained by population structure analysis was not related to their geographical distribution. For example, pathotypes 117-2, 21, and 184 belonged to the North India region while population structure analysis classified them in SP1, SP2, and admixture, respectively. Our results comprehend that the *Pgt* population in the Indian subcontinent might have evolved from two clonal events, and subsequently, gene flow, long-distance migration, and somatic hybridizations could have contributed to the development of the admixture population, which is not exceptional in *Puccinia* spp. (Park et al., 1999; Park and Wellings, 2012). The low Nei’s genetic distance between different population combinations indicated that the *Pgt* population belonging to different geographical regions in the Indian subcontinent are closely related and thus explain poor genetic divergence. Similar findings are reported for two *P. striiformis* populations from Bajaur and Malakand in Pakistan, while substantial genetic divergence was observed between *P. striiformis* populations from Nepal, Pakistan, and Bhutan (Khan et al., 2019). Likewise, genetic divergence among *P. triticina* isolates collected from *Aegilops speltoides*, durum, and bread wheat; delimited by host specificity was reported from Israel (Liu et al., 2014). Genetic drift, gene flow, accumulated mutation, adaptation, and host specificity are suggested as the driving forces behind genetic divergence among *Puccinia* spp. (Kohn, 2005; Kolmer et al., 2020).

## Conclusion

The repository of 29 *Pgt* pathotypes collected during the last ∼90 years exhibited significant variability for virulence phenotype and SSR genotypes. The SSR markers used in this study were highly informative in explaining the genetic diversity among *Pgt* pathotypes. As revealed by different statistical programs and hypothetical evolutionary hierarchy, the gene flow, long-distance dispersal, and single-step mutation for gain and/or rare loss of virulence could have contributed to cryptic genetic variation and evolution in the *Pgt* population from the Indian subcontinent. The findings of population structure and genetic diversity analysis of *Pgt* population, supplemented by deployment of effective Sr genes (*Sr26, Sr27, Sr31, Sr32, Sr33, Sr35, Sr39, Sr40, Sr43, SrTmp*, and *SrTt3*) in combination with other APR genes, could be utilized for strategic wheat stem rust management.

## Data Availability Statement

The original contributions presented in the study are included in the article/Supplementary Material, further inquiries can be directed to the corresponding author.

## Author Contributions

PP, SS, and SB conceived the idea and designed the experiments. PP, RT, and SK performed the virulence phenotyping. PP and RT performed SSR genotyping. PP, SS, OG, CL, and SA performed the data analysis and prepared first draft of the manuscript. PP and SB reviewed and revised the manuscript. All authors contributed to the article and approved the submitted version.

## Conflict of Interest

The authors declare that the research was conducted in the absence of any commercial or financial relationships that could be construed as a potential conflict of interest.

## Publisher’s Note

All claims expressed in this article are solely those of the authors and do not necessarily represent those of their affiliated organizations, or those of the publisher, the editors and the reviewers. Any product that may be evaluated in this article, or claim that may be made by its manufacturer, is not guaranteed or endorsed by the publisher.
